# Bosworth Dislocation without Associated Fracture

**DOI:** 10.1155/2018/7284643

**Published:** 2018-04-01

**Authors:** Austin D. Williams, Matthew Blue, Christian Douthit, Cyrus Caroom

**Affiliations:** Department of Orthopaedic Surgery and Rehabilitation, Texas Tech University Health Sciences Center, 3601 4th St. MS 9436, Lubbock, TX 79430, USA

## Abstract

One of the rarest ankle injuries is the Bosworth fracture-dislocation, whereby the distal fibula fractures and is lodged behind the tibia and is often unable to be reduced in a closed fashion. Even more rarely, a Bosworth dislocation without any accompanying fractures may occur. In this case, a 19-year-old male presented with a Bosworth dislocation, with the ipsilateral tibia having previously undergone intramedullary nailing. After closed reduction was attempted, open reduction and fixation was performed, directly reducing the fibula and fixing the unstable syndesmosis with 2 quadricortical screws. Bosworth injuries are rare, yet severe, and should be treated in a timely manner. We were able to provide good reduction and fixation without requiring removal of the intramedullary nail, and we support the use of 2 quadricortical screws as a valid treatment option for the fixation of Bosworth dislocations.

## 1. Introduction

Bosworth fracture-dislocations are a rare occurrence [1, 2]. This occurs when the foot is placed in extreme external rotation, at which point the distal portion of the fibula fractures, dislocates, and is impinged behind the posterior tibial tubercle [2, 3]. When this occurs, the fracture is almost always unable to be reduced in a closed fashion and must be treated with open reduction and internal fixation to prevent articular damage as a result of repeated attempts at closed reduction [3–5]. In this report, we describe a Bosworth dislocation of the fibula without an associated fracture, a situation described only a handful of times in the literature [1, 2, 6], and emphasize the importance of early operative reduction in minimizing unnecessary closed reduction attempts.

## 2. Case Presentation

**Figure 1:**
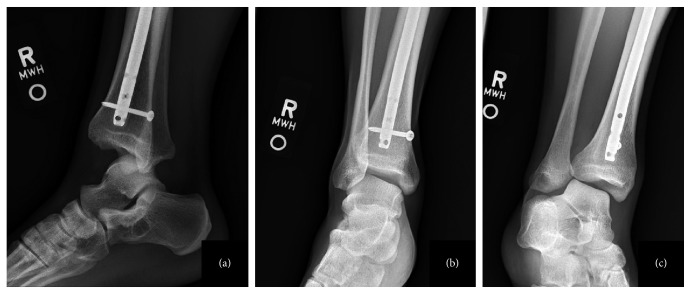
Preoperative lateral (a), AP (b), and mortise (c) radiographs showing tibiotalar dislocation and impingement of the fibula behind the tibia with no fractures.

**Figure 2:**
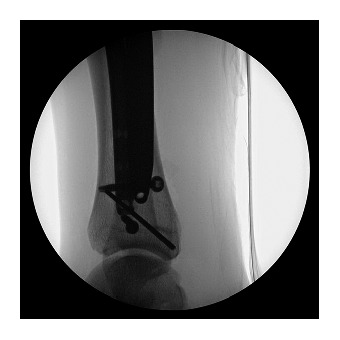
Intraoperative lateral radiograph showing placement of syndesmotic screws and the undamaged previously placed tibial nail.

**Figure 3:**
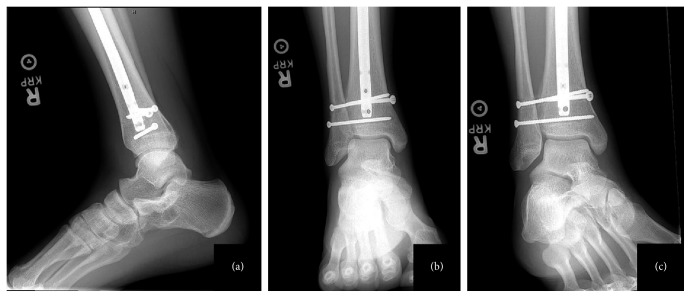
Postoperative lateral (a), AP (b), and mortise (c) radiographs showing 2 quadricortical screws stabilizing the syndesmosis with no evidence of hardware failure.

A 19-year-old male with a history of a right tibial stress fracture with intramedullary nail fixation 14 months before presented to the emergency department after jumping into a shallow lake and immediately suffering severe right ankle pain and inability to bear weight. He had had the proximal intramedullary nail screw removed 3 months prior to presentation due to discomfort but reported no other medical problems. On exam, there was significant swelling of the ankle, with global tenderness to palpation. The fibula was noted to be displaced, as palpation of the lateral aspect of the ankle resulted in contact with the tibia, and palpation at the normal region of the peroneal groove revealed the distal fibula. Motion of the ankle was limited in all planes; however, the foot was neurovascularly intact and sensitive to light touch. A 3-view X-ray of the ankle confirmed the dislocation of the fibula posterior to the tibia and posterior dislocation of the talus, while showing the stable intramedullary nail and distal screw with no apparent lucencies (Figure 1). Additionally, AP and lateral views of the entire tibia and fibula did not reveal any fractures, and the patient did not complain of any discomfort or tenderness to palpation outside of the ankle. Closed reduction was attempted in the emergency department; the talus was relocated, but the fibula was lodged in place and unable to be reduced. Understanding that repeated attempts at closed reduction most likely be unsuccessful and to minimize the risk of articular damage, iatrogenic fracture, or skin breakdown, the decision was made to proceed with open reduction. The patient consented to open reduction and internal fixation of the fibular dislocation and was taken to the operating room the same day. The time that elapsed from injury to operation start was just under 5 hours. From the time the patient had been seen in the emergency department to operation start was under 3 hours. A lateral incision was made, and the fibula was exposed, positioned behind the tibia. At this point, a hemostat was placed between the fibula and tibia and used as a lever to free the fibula. The fibula was reduced in an open fashion but noted to be unstable due to syndesmotic ligament disruption. The fibula was then held in place with a Kirschner wire and fixed by placing two quadricortical screws through the tibia, using radiographic guidance to avoid the previously placed nail and remaining distal screw (Figure 2). Following this, the syndesmosis was noted to be stable, and the incision was irrigated and closed. At 3 months postoperatively, the fixation continued to remain stable (Figure 3). The patient underwent removal of the syndesmotic screws 4 months postoperatively, and at 6 months from the initial injury, the patient reported mild pain with activity and had full strength but was limited to approximately 10 degrees of plantar flexion. His AOFAS Ankle-Hindfoot score was noted to be 85.

## 3. Discussion

This case is a rare variant of an already rare phenomenon. We are aware of 67 reported cases of Bosworth fracture-dislocations in the literature [2, 4]. Of these, 12 were dislocations without fracture, and 8 of those occurred in skeletally mature adults [1, 2]. The mechanism of an intact fibula trapped behind the tibia was first described in 1922 by Ashhurst and Bromer, who referenced a description by Huguier in 1848 [6]. Bosworth himself described the typical case of fibular impingement with fracture in 1947 [7]. Since then, published reports of this injury have been few and far between. To our knowledge, this is the first instance of a patient with adjacent hardware having a Bosworth dislocation. While quite rare, it is important to be able to recognize and treat this injury to minimize future sequelae. Delays in treatment may lead to poor healing, arthritic changes, and even compartment syndrome [8–11]. However, treatment may be delayed to account for management of soft-tissue swelling [12], but this should not postpone operative treatment for an excessive period [13]. In Downey et al.'s paper [2], the 2 patients in their series who underwent operative fixation at 1 and 10 days after injury suffered no sequelae, while the 3 patients who underwent surgery at 12–14 days had residual pain, even though 1 of those was fully compliant with the prescribed treatment regimen. For our patient, there appeared to be minimal soft-tissue damage at the time of evaluation, and we believed that urgent reduction would lead to a better outcome, and so we proceeded with operative management immediately after closed reduction was unsuccessful. It is important to be aware that the vast majority of Bosworth dislocations are unable to be reduced closed and that repeated attempts at closed reduction can cause subsequent problems [5]. This is why we quickly made the decision to proceed with operative reduction rather than risk further injury. Additionally, Downey et al. [2] also demonstrated improved results by using 2 quadricortical screws for syndesmosis fixation, as well as patient compliance with treatment programs. In this case, we also used 2 quadricortical screws and achieved good fixation of the syndesmosis, without any hardware failure in the adjacent tibia. The use of these screws appears to be a valid option in the fixation of syndesmotic injuries after a Bosworth dislocation. In addition, the decision was made to retain the patient's intramedullary nail at this point in time, even though it would allow easier syndesmotic fixation, as the implant was causing no discomfort to the patient and it would require the use of additional incisions and soft-tissue trauma. It could be theorized that the presence of the intramedullary nail in the ipsilateral tibia played a role in this injury, whether in the initial impact or during dislocation of the fibula, but unfortunately, such an effect cannot be determined specifically for the patient presented. However, further focus on injury patterns concerning intramedullary nails may be able to shine light on this potential interaction.

## 4. Conclusion

Bosworth fracture-dislocations are a rare and severe injury that must be recognized and evaluated in an expedient manner. Understanding that timely operative reduction of these injuries minimizes the risk of further injury is paramount, and no more than one closed reduction attempt should be made before proceeding to the operating room. This case shows support for the fixation of the unstable syndesmosis after reduction with 2 quadricortical screws, showing that this fixation can be performed safely if there is hardware in the ipsilateral tibia.
